# Supplementary material for: Chromothripsis during telomere crisis is independent of NHEJ, and consistent with a replicative origin
Source: Genome Res. 2019 May;29(5):737–49. doi: 10.1101/gr.240705.118 (PMC6499312; doi:10.1101/gr.240705.118)
Supplement: Supplemental Material [file supp_gr.240705.118_Supplemental_file_1.zip › contigs/annotated_contigs/DB54/contig.2.DB54_length_696_mean_cov_13.4454022989.docx]

**DB54_length_696_mean_cov_13.4454022989**

```
GGCCTGGGAAGAGGCAGGTGTGATGTGTGGGATTGAGACATGTCCAGGCACAGGAACCAATGAAAGAACTGTACAACATTTACTTTTTC
 >chr16:369647-370067 - E=6e-241
AGAGGGAGGAAATGGAAAGCAAAAAATACCCCAAAGAAGGCAAGAAAGAGCAAAAGGAAGTAGAGCAGATGTGATAAACCAACCCGATC

CGACGGTGGCCCAGCTCCCTCCACATCACACTAAATGTAACCGGACCAGACAGCCTCATTAAAAGGAAAAATTGTTATGCTACCTTTTT

AAAAGGCAGGCTGCTTACACAAGTAGAAGAATATAGAATGGTTAAATGTGAAAAGACGGAAGATGAAAGTACCACAGTGACCAGAGAGC

CGGTGCCGTGGCCATGCTGGATGTGGGAGCAGCCGCGGGAGTCCCAGAGAGGTGCCTTCCTGGG|TTAGCTGGGCACGGAGGCATGTGC
                                                                  >chr16:496590-496867 -
CTGTAATCCCAGCTACTCGGGAGGCTGAGGCAGGAGAATTGCGTGAACCAGGACTAGGAGGCGGAGGTTGCAGTGAGCTGAGATCACGC
 E=5e-150
CACTGCACTCCAGCCTGGGCGACAGAGCGAGAGTGCGTCTCAAAAAAAAACAAAAAAGTTTGAGATATATGAATAAAAAGGACTATATG

GAAATAAGTCCTCACACTGAAGTGTGCTTGGAAAACAGGTCAACATGTCTCTGTAGGACACATCTGGACACCAG
```
